# Isolated Increased Nuchal Translucency in First Trimester Ultrasound Scan: Diagnostic Yield of Prenatal Microarray and Outcome of Pregnancy

**DOI:** 10.3389/fmed.2021.737936

**Published:** 2021-10-18

**Authors:** Kyra E. Stuurman, Marjolein H. van der Mespel-Brouwer, Melanie A. J. Engels, Mariet W. Elting, Shama L. Bhola, Hanne Meijers-Heijboer

**Affiliations:** ^1^Department of Human Genetics and Amsterdam Reproduction and Development Research Institute, Amsterdam UMC, Vrije Universiteit Amsterdam, Amsterdam, Netherlands; ^2^Department of Clinical Genetics, Erasmus MC, University Medical Center Rotterdam, Rotterdam, Netherlands; ^3^EchoXpert, Amsterdam, Netherlands; ^4^Department of Human Genetics, Amsterdam UMC, Universiteit van Amsterdam, Amsterdam, Netherlands

**Keywords:** increased NT, normal karyotype, chromosomal microarray, prenatal diagnosis, fetal ultrasound scan

## Abstract

**Background:** Increased nuchal translucency (NT) is associated with aneuploidy. When the karyotype is normal, fetuses are still at risk for structural anomalies and genetic syndromes. Our study researched the diagnostic yield of prenatal microarray in a cohort of fetuses with isolated increased NT (defined as NT ≥ 3.5 mm) and questioned whether prenatal microarray is a useful tool in determining the adverse outcomes of the pregnancy.

**Materials and Methods:** A prospective study was performed, in which 166 women, pregnant with a fetus with isolated increased NT (ranging from 3.5 to 14.3 mm with a mean of 5.4 mm) were offered karyotyping and subsequent prenatal microarray when karyotype was normal. Additionally, all ongoing pregnancies of fetuses with normal karyotype were followed up with regard to postnatal outcome. The follow-up time after birth was maximally 4 years.

**Results:** Totally, 149 of 166 women opted for prenatal testing. Seventy-seven fetuses showed normal karyotype (52%). Totally, 73 of 77 fetuses with normal karyotype did not show additional anomalies on an early first trimester ultrasound. Totally, 40 of 73 fetuses received prenatal microarray of whom 3 fetuses had an abnormal microarray result: two pathogenic findings (2/40) and one incidental carrier finding. In 73 fetuses with an isolated increased NT, 21 pregnancies showed abnormal postnatal outcome (21/73, 28.8%), 29 had a normal outcome (29/73, 40%), and 23 were lost to follow-up (23/73, 31.5%). Seven out of 73 live-born children showed an adverse outcome (9.6%).

**Conclusions:** Prenatal microarray in fetuses with isolated increased NT had a 5% (2/40) increased diagnostic yield compared to conventional karyotyping. Even with a normal microarray, fetuses with an isolated increased NT had a 28.8% risk of either pregnancy loss or an affected child.

## Introduction

One of the techniques used in prenatal diagnostic testing is chromosomal microarray (array comparative genomic hybridization, CGH or single nucleotide polymorphism, SNP array). Before prenatal microarray was available, chromosomal karyotyping was the standard technique to perform when soft markers or structural anomalies were seen on fetal ultrasound scan. With the introduction of chromosomal microarray, a higher resolution of the genome can be achieved compared to conventional karyotyping, and therefore, a prenatal microarray is nowadays used as a standard tool if structural anomalies are seen on fetal ultrasound scan. Studies have shown that prenatal microarray for a wide range of abnormal ultrasound findings increases the percentage of genetic abnormalities detected by ~5–17% when compared to standard karyotyping (1–3). In our hospital, we implemented prenatal array CGH in 2011 first in pregnancies in fetuses with an increased nuchal translucency (NT) without additional abnormalities on a first trimester ultrasound (dating scan or scan in the context of first trimester combined screening), and who had a normal karyotype with standard karyotyping or Rapid Aneuploidy Detection through Quantitative Fluorescence-PCR (QF-PCR).

Nuchal translucency is defined by the translucent area in the neck region of the developing fetus, which can be visualized by ultrasound between 11 and 13 + 6 weeks of gestation. Isolated increased NT is defined as an NT being the sole anomaly without any other soft markers or structural defects visible on fetal ultrasound. Worldwide, NT measurement was used for the first trimester combined screening of trisomies 21, 18, and 13 and monosomy X. An increased NT increases the risk of chromosomal aneuploidy (4). Overall, approximately half of the fetuses with an increased NT show an abnormal karyotype (5). The fetuses with normal karyotype are, however, still at increased risk for a wide variety of structural defects and genetic abnormalities (6–9), such as cardiac defects (10) and Noonan syndrome (11, 12). Some of these abnormalities may be explained by submicroscopic genomic deletions or duplications and therefore, can be detected only by prenatal microarray.

Many studies have reported data on the use of prenatal microarray in fetuses with (isolated) increased NT, with the first report starting as early as 2003 (13) and approximately five English studies in the years 2020 and 2021 (14–18). These studies show a relatively wide range (0–20%) of additional genetic findings in fetuses with increased NT with regard to prenatal microarray when compared to standard karyotyping. Additionally, isolated increased NT shows a lower diagnostic genetic yield than increased NT with additional structural defects on (second trimester) ultrasound scan (19). However, in the last years, non-invasive prenatal testing (NIPT) has been introduced and is offered to pregnant women for screening of trisomy 21, 18, and 13 as a first-tier test (20). As a result, first trimester combined screening is only performed in ~3% of pregnancies in the Netherlands at the moment (21). On the other hand, worldwide a first trimester ultrasound scan at 13 weeks of gestation is still appreciated as an important tool for early detection of possible fetal anomalies or genetic disorders (22, 23). In the Netherlands, from September 1, 2021 onward, first trimester ultrasound scan is offered to all pregnant women free of charge as part of the National Prenatal Screening Program. This scan will screen for growth and structural anomalies and will also include NT measurement. As a result of the implementation of this first trimester ultrasound scan, it is expected it will lead to more referrals to tertiary health centers.

Isolated increased NT on first trimester ultrasound is also associated with an increased risk of adverse outcome of the pregnancy. The rate of adverse pregnancy outcome is strongly correlated with the severity of the increased NT and the presence of additional anomalies on first- or second-trimester ultrasound scan (9, 24).

The aims of this study were 2-fold: (1) to assess the diagnostic yield of prenatal microarray in a cohort of fetuses with isolated increased NT, and (2) to assess the value of prenatal microarray by determining the outcome of pregnancy in a larger cohort of fetuses with isolated increased NT, such as fetuses in whom prenatal microarray was performed.

## Materials and Methods

### Patients

A prospective study was performed in which 166 women who had first trimester combined screening in one of the referral centers and showed an increased NT in the fetus were referred to the Amsterdam UMC, location VUmc for a prenatal invasive procedure. These women were routinely offered conventional karyotyping. Because QF-PCR was introduced at the Amsterdam UMC in 2010, 14 samples received QF-PCR in addition to karyotyping. When a normal karyotype in the fetus was confirmed and no other abnormalities on an early ultrasound scan (before 16 weeks of gestation) were seen, the patient was offered subsequent additional microarray analysis. Before performing microarray analysis, couples were counseled about the testing process, benefits, and limitations of testing and possible outcomes. Informed consent was signed and couples received an additional information letter. The local institutional ethics board approved the study.

The exclusion criteria were pregnancies in which teratogenic medication was used, monochorionic twin pregnancies, and women with significant underlying medical conditions.

Increased NT measurement was defined as equal to or >3.5 mm, and NT measurement was performed between 11 and 13+6 weeks of gestation, according to the Dutch Society of Obstetrics and Gynecology guidelines (25). Because NT measurements were performed at referral centers, the gestational age at the time of the measurement was not registered at our center.

### Samples

All samples from the dataset used for microarray analysis were received between January 1, 2011, and August 1, 2013, from the Department of Obstetrics and Gynecology at Amsterdam UMC, location VUmc. All, except for one, of the samples were chorion villi samples (CVS). One sample was amniotic fluid. This time interval was chosen because in this period, the use of prenatal microarray was first introduced and analyzed at our center.

Parental blood samples were simultaneously tested and interpreted to differentiate between potential familial and *de novo* pathogenic copy number variations (CNVs). In case DNA was isolated from cultured chorionic villi cells and a female fetus was concerned, DNA was tested for maternal contamination. In case parental blood samples were not received, microarray on the fetus was not performed, and the patient was excluded from the study.

### Postnatal Follow-Up

All ongoing pregnancies with isolated increased NT (with or without prenatal microarray) and normal second-trimester ultrasound scan (increased NT had resolved) presented at our hospital between January 2011 and August 2013 were followed up after birth. In the Netherlands, the first checkup right after birth is performed by a midwife when an uncomplicated birth has occurred. In case of a more complicated birth (e.g., Cesarean section), a pediatrician will perform the first check. When no problems occur, the children are followed up routinely by a doctor specialized in youth development according to the national guidelines. With regard to this study, the first author saw the children for the first time between 3 and 6 months after birth if earlier problems did not occur. They were checked for dysmorphic features and developmental parameters. One year and 4 years after the first visit, the children were evaluated again. In case of concerns, the children were evaluated earlier than at the regular intervals. The maximum follow-up time after birth was 4 years.

### Cell Culture and DNA Extraction

Samples were received in the laboratory for cytogenetic studies. Results of conventional karyotyping and QF-PCR were awaited first. In case of a normal result, microarray was subsequently performed on DNA extracted from a part of the sample. Patients were excluded from microarray when there was not enough fetal material to perform a subsequent microarray analysis.

DNA was extracted from uncultured cells using Wizard^Ⓡ^ (Promega, Madison, WI, USA) and from cultured cells using Qiagen BioRobot^Ⓡ^ (Qiagen GmbH, Hilden, Germany) according to the protocols of the manufacturer. Maternal contamination was tested with fragment analysis using PowerPlex16 (Promega, Madison, WI, USA).

### Microarray

Agilent CGH 180K oligo array (Agilent Technologies, Santa Clara, CA, USA; Amadid 023363) was used as an array platform and performed according to the instructions of the manufacturer. The overall median probe spacing of this platform was 13 kb. As reference DNA, a commercial reference pool of Kreatech, consisting of healthy men and women, was used (sex-matched experiments).

Data analysis was performed using Nexus Copy Number versions 5.0, 6.1, and 7.0, and interpreted using Cartagenia Bench 4.0 Feb-2012 (genome build hg18 and hg19).

Standard settings for CNVs in Nexus were applied: threshold for probe median: gain 0.3 and loss −0.3. Minimal probes for a call are 20 per segment. The interpretation of CNVs has been done according to the criteria as described previously (26). We analyzed trios to assess whether CNVs were *de novo* or inherited.

The microarray was considered normal if only benign class 1 or 2 CNVs were detected. The microarray was considered abnormal if a most likely clinically relevant (classes 4 and 5) CNV was found. Variants of unknown clinical significance (class 3) were discussed internally before reporting them.

### Statistical Analyses

The data were analyzed in SPSS V.22. For statistical analysis, descriptive statistics were used.

## Results

**Figure 1:**
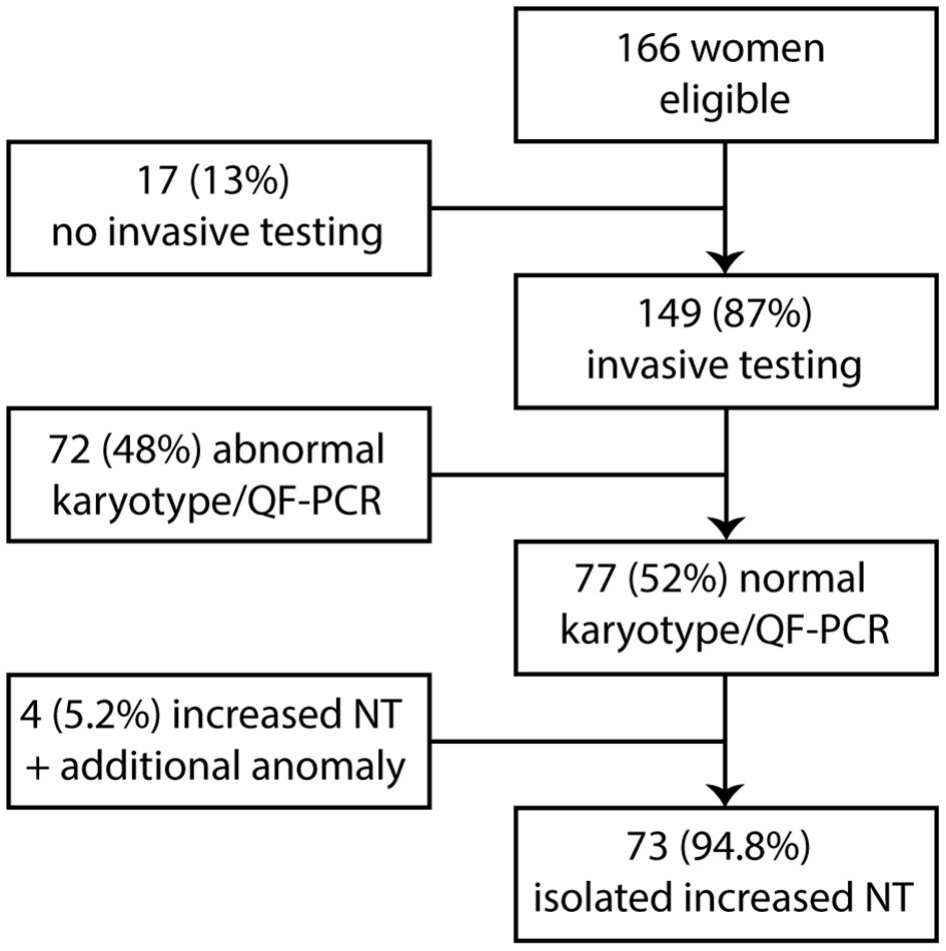
Eligible fetuses with increased NT and normal karyotype. NT, nuchal translucency.

**Figure 2:**
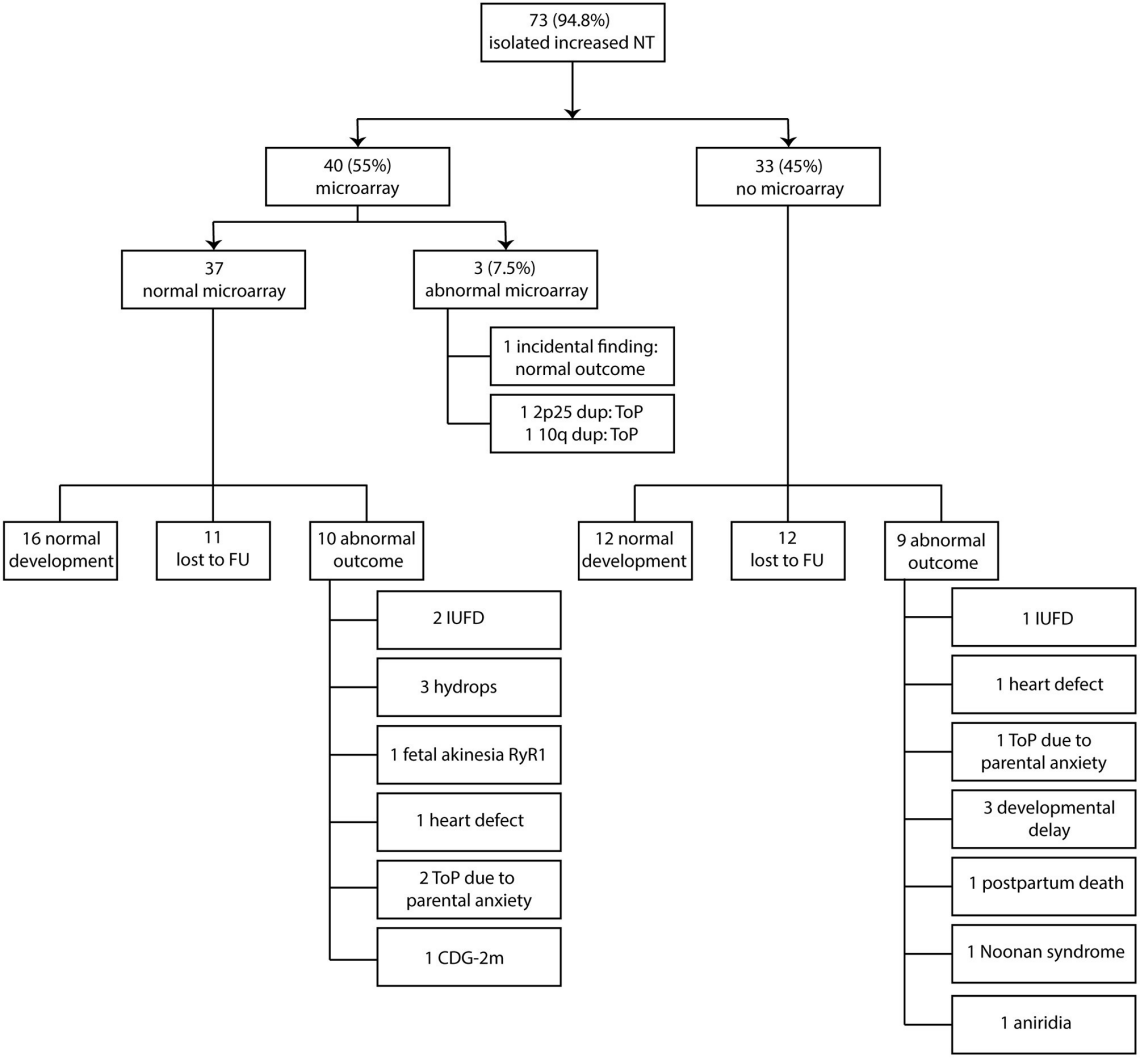
Clinical and genetic outcome in relation to prenatal microarray. NT, nuchal translucency; ToP, termination of pregnancy; FU, follow-up; IUFD, intrauterine fetal death; CDG-2m, congenital disorder of glycosylation type 2 m.

Between January 2011 and August 2013, 166 women were seen in our hospital because of increased NT in the fetus. The mean maternal age for all pregnancies with increased NT was 33.8 years (range 21–46 years). The mean thickness of increased NT of all pregnancies was 5.4 mm (range 3.5–14.3 mm). A total 149 of 166 women opted for invasive prenatal testing (87%) with standard karyotyping or QF-PCR. Total 77 of 149 fetuses had normal QF-PCR or karyotype results (52%). The mean NT thickness of these 77 fetuses was 4.8 mm (range 3.5–10.2 mm). The other 72 of 149 fetuses had a chromosome abnormality. Thirty-two fetuses had trisomy 21 (21.5%), 24 fetuses had trisomy 18 (16%), 4 fetuses had trisomy 13 (2.7%), 11 fetuses had 45,X (7.4%), and one fetus had 47,XXY (0.7%). Four of the 77 fetuses (5.2%) had an increased NT plus additional anomalies on an early ultrasound scan at ~14 weeks of gestation and were excluded from this study (Figure 1). The remaining 73 fetuses were eligible for microarray. The mean NT thickness was 4.7 mm (range 3.5–10.2 mm). On 40 of 73 fetuses, a prenatal microarray (55%) was performed. The mean NT thickness was 4.7 mm (range 3.5–9.3 mm). The remaining 33 fetuses did not receive a prenatal microarray due to an insufficient amount of DNA, no access to parental samples or microarray declined by the parents as a subsequent test (Figure 2). The mean NT thickness was 4.7 mm (range 3.5–10.2 mm; Table 1).

**Table 1:** Overview of NT measurement in relation to performed prenatal microarrays.

**NT measurement (mm)**	**Microarray: Yes, normal (*n*)**	**Microarray: Yes, abnormal (*n*)**	**Microarray: Not performed (*n*)**
3.5–4.4	21	1	21
4.5–5.4	8	0	4
5.5–6.4	7	1	4
≥6.5	2	0	4

*NT, nuchal translucency*.

### Diagnostic Yield of Prenatal Microarray

Two of the 40 fetuses (5%) that underwent microarray analysis had a likely pathogenic CNV and 1 fetus had incidental findings (2.5%). Of the two fetuses with pathogenic CNV, one had a 10.9 Mb duplication on chromosome band 10q25.1q26.12. Of note, this fetus had a normal second-trimester ultrasound scan; yet the pregnancy was terminated due to the pathogenic finding. The other pathogenic chromosome abnormality was an 8.2 Mb duplication on chromosome 2p25. This pregnancy was terminated before the second-trimester ultrasound scan was performed and further fluorescence *in situ* hybridization (FISH) analysis showed a paternally inherited unbalanced translocation between chromosomes 2 and 22. Both pathogenic CNV findings were not detected with standard karyotyping. In a third fetus, an incidental finding was identified: a 37 kb deletion on chromosome 15q26.1 was detected. In this deletion, the *RLBP1*-gene: (OMIM #180090) is located, which is involved in autosomal recessive diseases of the retina. The deletion was paternally inherited. The fetus did not show any anomalies on second-trimester ultrasound scan, and a healthy girl was born. We did not identify *de novo* variants of unknown significance (VUS) in the 40 fetuses that underwent microarray (Table 2 and Figure 2).

**Table 2:** Overview of CNVs found on prenatal microarray.

**Case**	**NT**	**Chromosome**	**Size and type**	**Categorization**
1	5.5	10q25.1–26.12	10.9 Mb duplication	Pathogenic
2	3.9	2p25	8.2 Mb duplication	Pathogenic
3	5.2	15q26.1	37 kb deletion	Incidental finding

*CNV, copy number variation; NT, nuchal translucency*.

### Pregnancy Outcome in Relation to Prenatal Microarray

#### The Outcome of Pregnancy in Fetuses With Normal Prenatal Microarray

Three of the 40 fetuses who underwent prenatal microarray had an abnormal result. Therefore, 37 fetuses had a normal prenatal microarray result. Sixteen fetuses had a normal outcome (16/37, 43%), 11 were lost to follow-up (11/37, 30%), and 10 had an abnormal outcome (10/37, 27%), independent of the second-trimester ultrasound scan. The fetuses with an abnormal outcome showed the following anomalies: two fetuses died *in utero*, three developed hydrops (two were terminated and one resulted in intrauterine fetal death), one developed fetal akinesia due to a postnatally through exome sequencing confirmed homozygous *RyR1* (OMIM #180901) pathogenic variant, and the pregnancy was terminated, and one had a severe congenital heart defect (situs ambiguus of the atria with left isomerism, double outlet left ventricle, unbalanced atrioventricular septal defect, hypoplastic right ventricle) on second-trimester ultrasound scan, and the pregnancy was subsequently terminated. With regard to the fetuses with hydrops, the following additional testing was performed: two of three fetuses received DNA analysis for RASopathies and lysosomal testing, which was normal in both. No further testing was carried out. The other fetus did not receive any additional testing due to parental choices. Two pregnancies were terminated in a private clinic on the request of parents due to parental anxiety on a poor outcome, and one fetus had a postnatal confirmed congenital disorder of glycosylation type 2m (CDG-2m; Figure 2).

### The Outcome of Pregnancy in Fetuses Without Prenatal Microarray

In the group of 33 fetuses without prenatal microarray, 12 showed normal postnatal development (12/33, 36%), 12 were lost to follow-up (36%), and 9 fetuses showed an abnormal (postnatal) outcome, independent of second-trimester ultrasound scan (9/33, 27%). The following anomalies with regard to abnormal outcomes were reported: one intrauterine fetal death, one with a congenital heart defect (tricuspid valve atresia), and the pregnancy was subsequently terminated. One pregnancy was terminated on the request of parents due to parental anxiety on a poor outcome. Three children showed developmental delay after birth, without an underlying (genetic) diagnosis at the time of last evaluation. In all three children, a postnatal microarray was performed and did not show any pathogenic CNVs. One of the children with developmental delay also had craniosynostosis. DNA analysis for a specific craniosynostosis syndrome was performed, and no pathogenic variants were found in the *FGFR1* (OMIM #136350), *FGFR2* (OMIM #176943), *FGFR3* (OMIM #134934), and *TWIST* (OMIM #601622) genes. A VUS was found in the *TCF12* (OMIM #600480) gene, which was also found in the healthy unaffected mother. One fetus was diagnosed with severe brain anomalies at 25 weeks of gestation and this pregnancy continued until term birth. The baby died several hours after birth, and additional postnatal chromosome microarray was normal. No other testing had been performed and no underlying (genetic) diagnosis was made at the time. One child had Noonan syndrome with a prenatally confirmed pathogenic *PTPN11*-gene (OMIM #176876) variant. One child had aniridia. DNA analysis performed on a buccal swab sample detected a mosaic *PAX6*-gene (OMIM #607108) duplication (Figure 2).

Both microarray and non-microarray groups taken together, in 73 fetuses with an isolated increased NT, 21 pregnancies had an abnormal postnatal outcome (12 in the microarray group that include the two pathogenic CNVs, 9 in the non-microarray group) (21/73, 28.8%), 29 had a normal outcome (including the fetus with the incidental findings) (29/73, 40%), and 23 were lost to follow-up (23/73, 31.5%). The abnormal postnatal outcome was defined as any event that prevented the birth of a healthy normally developed child, such as termination of pregnancy (including due to parental anxiety) or an affected child after birth.

In total seven live-born children showed adverse outcomes. The overall risk of having an affected child in pregnancies with isolated increased NT in the fetus is therefore 9.6% (7/73).

### Pregnancy Outcome in Relation to Second-Trimester Ultrasound Scan

#### Second-Trimester Ultrasound Scan in Fetuses With Prenatal Microarray

**Figure 3:**
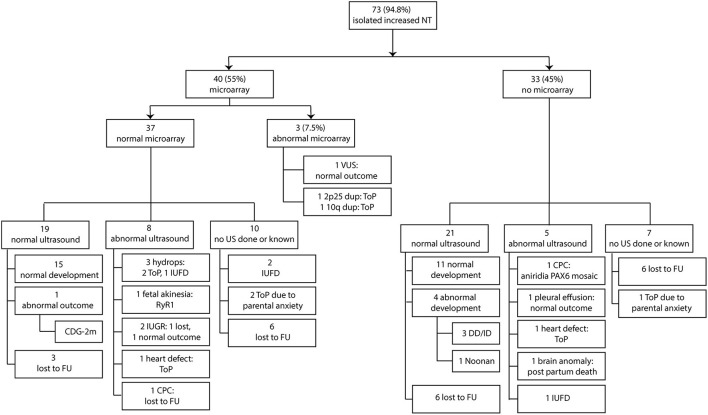
Clinical and genetic outcome in relation to second-trimester ultrasound scan. NT, nuchal translucency; ToP, termination of pregnancy; FU, follow-up; CDG-2m, congenital disorder of glycosylation type 2m; IUFD, intrauterine fetal death; IUGR, intrauterine growth restriction; CPC, choroid plexus cysts; DD/ID, developmental delay and intellectual delay; US, ultrasound scan.

In the group with 37 fetuses with normal prenatal microarray, 19 fetuses had a normal ultrasound scan (19/37, 51%) of which 15 had a normal outcome after birth (15/19, 79%), three were lost to follow-up, and one had an abnormal outcome (CDG-2m, 5%). Ten fetuses (10/37, 27%) were either lost to follow-up in the pregnancy or did not receive a second-trimester ultrasound scan due to intrauterine fetal death or termination of pregnancy due to parental anxiety about a poor outcome. Seven fetuses showed anomalies on the second-trimester ultrasound scan, which included hydrops development in three fetuses, fetal akinesia in one fetus, fetal growth restriction (FGR) in two fetuses, and a heart defect in one fetus. In addition, choroid plexus cysts (CPCs) were observed in an eighth fetus. As a result, the second-trimester ultrasound scan was not entirely normal in 8 of the 37 fetuses (22%). Two of the three fetuses with hydrops were subsequently terminated, the other died *in utero*. For two of three fetuses, DNA analysis for RASopathies and lysosomal testing was performed, which was normal in both. No further testing was carried out. The other fetus did not receive any additional testing due to parental choices. One of the fetuses with suspected FGR (in general defined as estimated fetal weight and abdominal circumference <p10 with a significant bending growth curve according to the International Society of Ultrasound in Obstetrics and Gynecology (ISUOG) Practice Guidelines) (27) on second-trimester ultrasound scan had actually a normal birth weight of 2,700 g and was healthy, and the other was lost to follow-up. The pregnancy of the fetus with the heart defect was terminated, and the one with choroid plexus cysts (CPC) was lost to follow-up (Figure 3).

### Second-Trimester Ultrasound Scan in Fetuses Without Prenatal Microarray

Twenty-one of 33 fetuses without prenatal microarray had a normal second-trimester ultrasound scan (21/33, 64%), of which 11 had a normal outcome after birth (11/21, 52%), 6 were lost to follow-up (6/21, 29%), and 4 had an abnormal outcome (global developmental delay in three, Noonan syndrome in one, 4/21, 19%). In the three children with neurodevelopmental delay, chromosomal microarray was performed when the delay became evident. The results were normal in all three. No specific clinical diagnosis was made in all three children and as a result, no further testing was performed at that time. Seven fetuses (7/33, 18%) were either lost to follow-up in the pregnancy or did not get a second-trimester ultrasound scan due to termination of pregnancy due to parental anxiety on a poor outcome. Five fetuses showed anomalies on the second-trimester ultrasound scan (5/33, 15%), which included one intrauterine fetal death, one heart defect, one fetus with pleural effusion and echogenic bowel, and one fetus with severe brain anomalies and one showed CPC. As mentioned earlier, the pregnancy with the heart defect was terminated. The fetus with pleural effusion and echogenic bowel had a normal outcome, the fetus with the brain anomalies died shortly after birth, and the fetus with CPC had postnatal aniridia (Figure 3).

Of all 73 fetuses with normal karyotype (without taking microarray into account), 40 fetuses showed a normal second-trimester ultrasound scan. Five of the 40 fetuses with a normal second-trimester ultrasound scan turned out to have an abnormal postnatal outcome (5/40, 12.5%).

Of note, two fetuses with second-trimester ultrasound anomalies (suspected FGR in one and pleural effusion and echogenic bowel in the other) had a normal outcome and were placed in the normal postnatal outcome group in the overall conclusion. Additionally, two fetuses with abnormal ultrasound scans (one with suspected FGR and one with CPC) were lost to follow-up after birth. Thus, if reported as abnormal outcome—e.g., an anomaly was seen on ultrasound scan—the risk of overall adverse pregnancy outcome increased to 34% (25/73 fetuses).

There was an unusual high percentage of follow-up loss in all groups. All pregnant women and live-born children who were lost to follow-up were first invited to the clinic by regular mail. If no reply came or a no show appeared, the pregnant women or parents in case of live-born children were contacted through phone. When no answers came after multiple tries, the patient was placed in the lost to follow-up category.

## Discussion

The first aim of this study was to gain more insight into the diagnostic use of prenatal microarray in fetuses with isolated increased NT and normal karyotype and/or QF-PCR and to evaluate the outcome of these fetuses. The results show that prenatal microarray increased the diagnostic yield in this group of patients with 5% (2 of 40 performed microarrays).

More than 30 studies have been published on the diagnostic yield of prenatal microarray in fetuses with an increased NT since Brisset et al. (13) were the first to report on this topic. A few of the earliest studies on prenatal microarray did not detect any submicroscopic deletions or duplications (28, 29), but the authors used low-resolution microarrays. Therefore, smaller deletions and duplications might have been missed. Other studies, such as the one from Lund et al. (30) reported a much higher diagnostic yield of 12.8% compared to our study. The explanation of author for this higher rate is the use of a high-resolution prenatal microarray (50 kb). However, in our lab, the same platform was used and we report a much lower diagnostic yield. Although Lund et al. commented on including fetuses with increased NT without other anomalies on the NT-ultrasound scan, it is unclear if a detailed follow-up scan at a later gestational age showed abnormalities in these fetuses. If so, this might explain their higher rate of pathogenic findings. In 2015, Grande et al. (19) reviewed all published papers on fetuses with an increased NT and established an overall 5% diagnostic yield for increased NT as a sole finding and a 7% yield for fetuses with increased NT and associated anomalies. Our percentage of pathogenic findings is therefore inline with most other studies (19).

In our cohort, two pathogenic CNVs were detected. The first one we identified was a 10.9 Mb duplication on chromosome segment 10q25.1q26.12. Yunis and Sanchez first described the 10q duplication syndrome in 1974 (31). Pure trisomy 10q24 → qter anomalies are characterized by pre- and postnatal growth restriction, severe intellectual disability, and structural defects of the heart, eyes, kidneys, and lower limbs. More distal trisomy anomalies starting from 10q25 have a less severe phenotype and lack major structural anomalies (32–35). Our case resulted in a termination of pregnancy after a second-trimester ultrasound scan was performed and no anomalies were identified. In the second case, the 8.2 Mb duplication on chromosome 2p25 was shown to be due to unbalanced translocation between chromosomes 2 and 22. Because of the large number of repetitive sequences and the absence of probes for the p-arm on chromosome 22, a small deletion on this chromosome was not picked up with prenatal microarray. However, additional standard karyotyping and FISH showed an unbalanced translocation. The translocation was paternally inherited. 2p25 duplications have not been well-defined, but are expected to cause structural anomalies and intellectual disability (36–39). These two pathogenic CNVs could not be picked up with conventional prenatal karyotyping, and therefore microarray has an added value to the first-tier tests. Thus, the results from our cohort confirm the added relatively low diagnostic yield of 5% in fetuses with an increased NT. However, it remains debatable whether performing additional prenatal microarray is worthwhile in fetuses with isolated increased NT. In the review of Grande et al. (19) (1,695 pregnancies in 17 studies), the same 5% diagnostic yield in fetuses with an isolated increased NT is reported. However, one can easily justify performing a prenatal microarray in fetuses with increased NT as invasive prenatal testing is performed for lower risks, such as testing in a pregnancy in which parents have a previous child with a random *de novo* pathogenic variant. Therefore, prenatal microarray in fetuses with isolated increased NT and normal Rapid Aneuploidy Detection is justified and aligns with the growing trend to offer this test to all patients with (isolated) increased NT and to provide the counseling so the patient can make a well-informed decision.

The second aim of our study was to gain insight into the development of children who presented with an isolated increased NT in pregnancy and to what extent prenatal microarray adds value in terms of predicting their outcome. Although we had a relatively high percentage of pregnancies and children who were lost to follow-up, we identified an overall percentage of 28.8% in an adverse outcome in fetuses with normal karyotype, such as terminations of pregnancy due to hydrops, intrauterine fetal demise, structural defects, and the adverse outcome after birth. Specifically, the overall percentage of adverse outcomes in all live-born children was ~9.6%. These percentages of adverse outcomes in fetuses with increased NT and normal karyotype and their outcome in live-born children are in agreement with several other studies (7, 40, 41). Additionally, the children who were born and presented with developmental problems all had an underlying diagnosis that could not have been picked up with prenatal microarray. Furthermore, the case with Noonan syndrome would not have been detected prenatally with the suggested criteria from Croonen et al. (11), because the NT in this fetus had resolved and a detailed second-trimester ultrasound scan did not identify additional anomalies specific for Noonan syndrome. However, Noonan syndrome would have been diagnosed with the recommendations from Stuurman et al. as every fetus with an isolated increased NT above 5.0 mm is eligible for RASopathy testing (12).

The percentage of live-born children with an adverse outcome was not altered as a result of performing prenatal microarray. Prenatal microarray only shows a relatively low additional diagnostic yield in fetuses with isolated increased NT and the live-born children in this study who did not receive a prenatal microarray but were eligible for microarray postnatal, did not have an abnormal postnatal microarray result. Therefore, the question is raised whether prenatal whole exome sequencing (WES) would be a suitable additional test in fetuses with an isolated increased NT. The first studies on prenatal WES suggested a 10–25% increase in diagnostic yield in fetuses with various sonographic abnormalities (42–44). Approximately six studies on prenatal WES have included fetuses with an isolated increased NT and normal microarray results (16, 42, 45–48). The diagnostic yield of prenatal WES in these studies varies from 0 to 13% with an average of 5%. In our cohort of fetuses, the CDG-2m, *RyR1*-related myopathy, Noonan syndrome, and possibly the mosaic PAX6 duplication might have been detected with prenatal WES. However, WES in fetuses with isolated increased NT warrants a careful approach as there is a substantial risk of unsolicited pathogenic findings that might not be related to the ultrasound findings (e.g., hereditary arrhythmias or hereditary cancer) and can cause anxiety in the future parents. Additionally, a prenatal phenotype is different from a postnatal phenotype, and it might be complicated to interpret variants of unknown significance (VUS) for their pathogenicity. In the Netherlands, therefore, these VUS are not reported back during pregnancy at the moment. Resolved edema is a favorable prognostic factor in the outcome (49), so the timing of offering prenatal WES can be important as well.

It is interesting to note that only two of the affected children had an abnormal second-trimester ultrasound scan. A second-trimester ultrasound scan is an important tool in the evaluation process but may give false reassurance as is seen in our cohort.

Like other studies in the field, our patient group was small as well for the outcome of prenatal microarray as for the outcome of follow-up. It also lacks long-term follow-up and a control group. Additionally, there was a high percentage of pregnancies and children that were lost to follow-up.

In conclusion, the prenatal microarray is of small but an added value (5%) as a diagnostic test to identify (submicroscopic) chromosomal anomalies in fetuses with an isolated increased NT and should be offered as standard clinical practice. However, even if the result of first trimester microarray was normal, in our study isolated increased NT thickness was still associated with a 28.8% (21/73) risk of pregnancy loss (spontaneous or induced) or an affected child. There remains an increased and not negligible risk for an adverse outcome in live-born children that had an isolated increased NT (7/73, 9.6%). In the near future, prenatal WES might be offered more frequently, preceded with genetic counseling.

## Data Availability Statement

The original contributions presented in the study are included in the article/supplementary material, further inquiries can be directed to the corresponding author/s.

## Ethics Statement

The studies involving human participants were reviewed and approved by Local Institutional Ethics Board of the Amsterdam University Medical Center, Vrije Universiteit (METC). Written informed consent to participate in this study was provided by the participants' legal guardian/next of kin. Written informed consent was obtained from the minor(s)' legal guardian/next of kin for the publication of any potentially identifiable images or data included in this article.

## Author Contributions

KS and HM-H: conceptualization. MM-B and SB: formal analysis. KS: investigations. MAJE and KS: resources. MWE: supervision. KS: writing—original draft. KS, MWE, MAJE, SB, and HM-H: writing—review. All authors contributed to the article and approved the submitted version.

## Conflict of Interest

MAJE is currently employed by EchoXpert, but was employed by Amsterdam UMC, Vrije Universiteit when the research was conducted. The remaining authors declare that the research was conducted in the absence of any commercial or financial relationships that could be construed as a potential conflict of interest.

## Publisher's Note

All claims expressed in this article are solely those of the authors and do not necessarily represent those of their affiliated organizations, or those of the publisher, the editors and the reviewers. Any product that may be evaluated in this article, or claim that may be made by its manufacturer, is not guaranteed or endorsed by the publisher.
